# Supplementary material for: Are carbon emissions trading and green financial instruments synergistic? -Comprehensive quantitative research based on content analysis
Source: PLoS One. 2024 Mar 7;19(3):e0298601. doi: 10.1371/journal.pone.0298601 (PMC10919601; doi:10.1371/journal.pone.0298601)
Supplement: S1 Data — (ZIP) [file pone.0298601.s001.zip › Data/地级市Z_F工作报告.zip!/╒π╜¡/╓█╔╜╩╨/└┤╫╘╣1⁄2╓┌║┼í╛┬φ┐╦╩2╛▌═°í┐_2014.docx]

各位代表：

现在，我代表市人民政府向大会报告工作，请予审议，并请政协委员和其他列席会议的同志提出意见。

一、2013年的主要工作

2013年是实施新区发展规划的开局之年。面对严峻的宏观经济环境和新区起步阶段繁重的建设任务，我们坚持以科学发展观为指导，认真贯彻省委、省政府和市委的决策部署，在市人大、市政协的监督、支持下，勇担使命，四干兴舟，全市实现地区生产总值930.85亿元，增长8.5%；财政总收入137.4亿元，其中公共财政预算收入92.6亿元，分别增长3%和8.3%；城镇居民人均可支配收入和渔农村居民人均纯收入达到37646元和20573元，分别增长10%和10.6%；实现社会消费品零售总额331.7亿元，增长14.1%。

这一年，《浙江舟山群岛新区发展规划》获国务院批复；新区管委会挂牌成立；省委省政府召开新区建设动员大会，出台推进新区建设的若干意见，批准实施《浙江舟山群岛新区建设三年行动计划》；舟山港综合保税区通过国家验收并封关运作；部省际联席会议制度批复建立。这一年，我们把握新区谋发展、打基础、强后劲的阶段性特征，既立足当前，又着眼长远，修订完善新区（城市）总体规划、土地利用总体规划及一批专项规划；主动谋划战略性举措；积极申报建立自由贸易港区；大胆改革新区行政体制，强化新区背景下的政府管理创新和执行能力建设。这一年，我们仍留有不少遗憾：地区生产总值、外贸进出口等指标没有实现预期目标；新区大开发大建设的氛围还不够浓、速度还不够快，重大项目引进建设的突破力度还不够大；一些政府工作人员的激情、能力和作风与新区加快发展的要求还不相适应。

回顾总结一年来的工作，主要有以下几个特点。

（一）保稳促调有新成效。针对支柱产业持续低迷、企业生产经营困难的严峻形势，以产业转型和企业帮扶为重点，打好“组合拳”，认真调研并制定实施了船舶、航运、外贸和水产等专项扶持政策，实现规模以上工业增加值264.5亿元。推进船舶与海工示范基地建设，做大做强船舶龙头和特色优势企业，规模以上船舶工业产值增长8.8%，船舶工业新接订单增长84.5%。港口物流业加快发展，港口货物吞吐量达到3.14亿吨，增长7.9%。建成国家旅游服务标准化示范区，蜈蚣峙等区域管理水平进一步提升，全市接待游客突破3000万人次，总收入超过300亿元，分别增长10.7%和12.5%。远洋渔业发展势头良好，新增远洋渔船50艘，鱿鱼等水产品内销取得积极进展。渔农业实现总产值188.8亿元，增长15.4%。培育新兴产业，海工装备制造、海洋电子信息、通用航空产业发展起步，舟山成为全国海水淡化产业两个试点城市之一。积极推进“个转企”“小升规”等工作。深入开展“营改增”试点，累计为企业减少税负1.5亿元。

（二）改革创新有新突破。围绕“三强三优”创新行政体制，市政府工作部门、市级事业单位分别精简26.5%和25.1%；完善了新城、海洋产业集聚核心区、普陀山-朱家尖、金塘、六横等五个功能区的体制机制，激发发展活力；建立了市场统一监管、综合行政执法、海上联合执法的新机构。围绕“减、简、放”，改革审批制度，制定实施投资项目预审批、竣工联合验收等办法，在海洋产业集聚区、金塘、六横等功能区实行“零审批”“零收费”试点。开展了以集体资产股份制改革和宅基地跨村跨镇置换为主要内容的渔农村综合改革。探索战略性资源改革，在全国率先实行岸线、海岛、海域的统一收储，国土资源改革试点、区域用岛试点深入推进，海域使用“直通车”及招拍挂办法正式实施。创新融资方式，新引进商业银行5家，海投集团完成融资44亿元，交投集团发行20亿私募债，金融机构融资总量余额达到2437亿元，比年初增加219亿元。加快舟山港综合保税区建设，《舟山港综合保税区管理办法》正式实施。新增口岸开放面积3.54平方公里，低空空域开放规划成功获批。海洋科学城核心区启动建设，全市新增省级企业研究院及高新技术企业研发中心6家，引进科技创业领军人才项目49个，省“百人计划”作用明显，新区高端紧缺人才引进工作扎实开展。

（三）扩大投资有新进展。狠抓项目建设，完成固定资产投资750亿元，增长20.6%。老塘山码头五期、220千伏舟北变工程等建成投用，大陆引水二期工程具备通水条件，国际邮轮码头、钓梁围垦二期基本建成，定马公路复线、官山大桥等重大基础项目顺利推进，浙江燃料乙醇、国电海上风电等重大项目获国家批复，六横-梅山疏港公路接线工程开工建设。惠生海工等重大产业项目建成投用，鼠浪湖矿砂中转、黄泽山油品储运、光汇石油储运、六横电厂等项目顺利推进。实施“机器换人”，工业技改投入达到96.7亿元，增长42.6%。强化招商引资的政策举措，主动对接重大项目，成功举办杭州、北京等重大招商推介活动，深入实施浙商回归、山海协作工程，实际到位市外注册资金93.8亿元，增长25.7%。

（四）城乡建设有新成绩。城市基础设施不断完善，首条快速公交专线建成运行，新城商务区三期等一批城建项目顺利实施。“三改一拆”强力推进，超额完成了省政府下达的目标任务；交通治堵取得阶段性成效，新建改建城市道路37公里，新增公共停车位5818个。新渔农村建设不断深入，建成美丽海岛精品（特色）社区25个，改造渔农村困难家庭危旧房643户，村庄环境综合整治率达到90%，新农村电气化目标全面实现。生态环境保护不断加强，新区发展规划环评获批，森林城市创建通过省级验收。关停高耗能、重污染企业12家，排污权有偿交易顺利开展。“四边三化”“两清”工作取得成效。市区空气质量优良率达到90.1%，PM2.5达标率达到93.4%。单位GDP能耗下降3.4%，能耗总量增速控制在4.7%，超额完成省政府下达的能源“双控”目标任务。

（五）社会发展有新进步。十方面为民实事项目全面完成，全市民生事业支出121.66亿元，增长26.8%，占预算支出64.1%。城镇登记失业率控制在2.7%，80%的低收入渔农户实现脱贫，城乡低保救助标准提高了13%。房地产市场保持稳定，建成保障性住房2448套，廉租房保障范围继续扩大。新增蔬菜基地2166亩，农贸市场提升三年计划全面完成。三个县（区）通过义务教育均衡发展评估，浙江海洋学院新校区启用，舟山职业技术学校成为国家级改革发展示范学校，公办中心幼儿园覆盖每个乡镇。文明城市创建活动扎实推进，海洋文化节成为全国性的节庆活动，新增渔农村文化礼堂52家，成功举办了系列海洋体育赛事。市级公立医院综合改革扎实推进，基本药物制度实施率达到92.5%，医疗救助“三统一”和城乡居民大病保险全面实施。率先实行了“单独二孩”生育政策。平安舟山建设深入推进，社会治安保持稳定，“铁桶固防”工程继续深化，打黑除恶、治理电信诈骗等专项行动深入开展，群众安全感满意率蝉联全省第一。普法、信访、调解、应急、流动人口服务管理等工作成效明显。加强公共安全和安全生产，气象、三防、森林防火、动植物防疫等工作不断深入；强力推进水产制冷行业整治，370家企业全部实施分类整治，已关停31家；渔船安全救助等信息系统建成投用，全市安全生产三项指标实现“零增长”。“网格化管理、组团式服务”继续深化。国防海防、民兵预备役、人民防空和双拥优抚等工作得到加强。民族、宗教、档案、史志事业不断发展，工青妇等群团组织作用进一步发挥，儿童、老龄、红十字、慈善和残疾人事业等取得新成绩。

一年来，我们自觉接受人大、政协和社会各界监督，政府系统承办的539件建议提案全部办结。注重发挥新区决策咨询委作用，建立了法律咨询专家组和“四位一体”法律顾问体系。加强廉政风险防控，强化政府性投资项目决策和审计监督，组建了造价中心、审计中心。坚决执行中央“八项规定”，推进预算决算公开和公务消费审计监督，“三公”经费支出减少12%，处理违反效能建设规定121人次。

各位代表，过去一年的成绩来之不易。这是省委、省政府和市委正确领导、全市百万军民共同奋斗的结果。在此，我谨代表市人民政府，向在座的各位人大代表、政协委员，向全市人民和建设者，向驻舟部队和省部属驻舟单位，向所有关心支持舟山发展的社会各界人士和海内外朋友们，表示衷心的感谢和崇高的敬意！

二、2014年工作总体要求和重点

当前，国际金融危机影响仍在延续，但世界经济已出现缓慢复苏态势；我国经济步入增长速度换档期，稳中求进、企稳向好的迹象明显，各项稳增长的政策效应逐步显现；通过近些年的不懈努力，新区蓄势待发、快速发展的基础日益扎实。基于对宏观形势和新区发展趋势的判断，确定今年工作的总体要求是，认真贯彻党的十八届三中全会精神，积极落实省委、省政府和市委工作要求，坚持稳中求进，突出“进中求快、快中求好”，以改革增活力，以开放促开发，以转型强后劲，以统筹惠民生，着力在加快发展上闯新路，在改善民生上出实招，在优化环境上下功夫，努力推动新区经济跨越发展、社会和谐稳定。

建议今年经济社会发展主要预期指标为：地区生产总值超1000亿元，增长10%以上；固定资产投资完成1000亿元，增长33%；公共财政预算收入突破100亿元，增长9%；城镇居民人均可支配收入和渔农村居民人均纯收入分别增长10%和11%。

重点抓好以下工作：

（一）强力扩大有效投资，推动经济快速发展。

加大招商引资力度。开展“招商引资和项目推进年”活动，研究出台招商引资考核管理办法，充分发挥县（区）和功能区的主战场作用，强化专业招商组的主力军地位，全市动员、领导带头、部门联动、全民参与，力争招引市外资金400亿元。海洋产业集聚区、金塘、六横等功能区要引进投资20亿元以上的项目，力争在引进50亿元以上的项目上有突破。不断创新招商引资方式，制定产业引导目录，加强项目策划和统筹协调，精心组织重大招商推介活动，深入实施浙商回归、舟商回归工程，完善“招大、引强、选优”机制，大力引进世界500强等优质外资项目。强化项目落地绩效激励，实施招商引资工作单列专项考核。优化招商引资环境，提供优质高效服务，营造“尊商重商亲商富商”的良好氛围。

推进重大项目建设。加快127个市级重点项目建设，实行领导干部“一对一”联系项目制度。推进一批重大产业项目，开工建设洋山国际物流中转园区一期、新奥LNG加注站、马岙港区液体化工品储运、光汇外钓石油储运及30万吨级码头等项目，加快富通电缆、永凯精制糖、册子实华码头二期、鼠浪湖矿砂中转等建设进度，完成黄泽山油品储运、中交三航海上风机等主体工程。加快推进一批“短平快”产业项目。抓好一批重大基础设施建设，开工建设秀山大桥，推进六横-梅山疏港公路接线工程、本岛南部滨海大道、329国道舟山段改建、大陆引水三期、钓梁围垦三期、小洋山围垦等建设，继续实施一批渔港海塘、航道锚地工程，确保柔性直流输电工程、六横电厂等建成投用，深化北向大通道、岱山北部围垦、500千伏大陆联网输变电工程等前期工作。抓好一批民生工程，加快旧城改造、安置房建设进度，基本建成大陆引水预处理及配水工程。

推动产业转型升级。加快发展临港先进制造业，力争规模以上工业增加值增长10.5%以上。大力扶持实体经济，培育龙头骨干企业，加快中小企业公共服务平台建设，激发民营经济发展活力。推进船舶、水产、塑机等行业的整合提升，积极发展海工装备、海洋电子信息、海洋生物医药等产业，建设集飞行保障、维修、驾培、制造为一体的通用航空产业基地。支持本地建筑企业做大做强。推进重大港航项目建设，促进货源本地化运输，拓展大宗商品加工交易，推动港工贸航联动发展。深化研究“江海联运”合作机制，开发长江航运市场。力争港口货物吞吐量达到3.45亿吨，争取“中国大宗商品交易中心”获国家批复。大力发展海洋旅游，完善综合配套，提升文化内涵，推进业态创新，开工建设观音文化园，推出一批禅修、民宿、运动休闲等特色项目，确保邮轮港正式运营，力争旅游人次达到3350万，总收入达到335亿元。积极推进金融、保险等服务业发展，培育文化创意、设计研发、服务外包等产业。建设东海油气开采后方服务和保障基地。抓好政务云工程和智慧医疗、智慧旅游等项目，促进重点产业“两化”融合和通信基础设施的共建共享，加快“无线岛城”“光网城市”建设。创新渔农业发展模式，培育家庭农场等新型经营主体，发展海岛特色生态渔农业，大力促进渔农业增效、渔农民增收。建设现代渔业体系，促进国内捕捞、海水养殖提质增效，推进国家远洋渔业基地建设，加快国际水产城改造，提升水产品精深加工水平。

（二）全力促进综保区建设，提高对外开放水平。

加快综保区高效运作。以本岛分区封关运作为契机，加强项目招引，力争注册企业达到1000家。完善管理运行体制，设立企业服务中心，3个工作日完成从项目核准到工商登记等手续。确保综保区码头正式开放。创新贸易监管模式，推进口岸“大通关”建设，实现“一次申报、一次查验、一次放行”，努力建成全国通关最便利的综保区之一。加快建设衢山分区，推进重大基础设施建设。主动接轨上海自由贸易试验区，探索建立合作机制。发挥综保区辐射效应，推进与海洋产业集聚区及其它经济功能区的联动发展。

探索综保区试行自由贸易政策。全面拓展保税燃料油加注业务，努力在保税燃料油调和混兑、外锚地加注、信息化监管等方面先行先试、创新突破，力争全年完成加注200万吨，并延伸发展船舶维修保养、生活补给等产业链，加快建设国际海事基地。发挥铁矿砂储运基地的优势，研究推进铁矿砂贸易。建立电商网购平台，探索开展跨境贸易电子商务业务。构建更为自由的投资服务体系，争取降低海洋产业投资准入门槛，努力把综保区打造成自由贸易港区的先行区。

争取设立舟山自由贸易港区。在综保区的基础上，继续深化舟山自由贸易港区建设总体方案，以大宗商品贸易自由化、海洋产业投资便利化和扩大现代海洋服务业对外开放为重点，探索扩大开放新模式。进一步完善机制，落实责任，只争朝夕，加强与国家有关部门对接汇报工作，力争早日获得国务院批复。

提升对外开放合作水平。进一步稳定出口、扩大进口，力争实现外贸进出口135亿美元，其中出口71亿美元，均增长7%。建设船舶出口基地，促进海产品外贸转型，发展国际物流服务外包。鼓励大宗商品、高档消费品、海洋装备零部件和水产品直接进口，推进浙台（普陀）经贸合作区建设。鼓励“走出去”境外投资，拓展国际营销渠道，开展境外渔业合作。深化与沪杭甬等长三角城市的合作交流，加快大小洋山规划建设步伐，推进北部港口与上海的融合对接，加快宁波-舟山港一体化进程。推进普陀山机场、惠群远洋渔业码头等口岸开放。

（三）着力加强改革创新，增强科学发展动力。

加快重点领域改革。坚决贯彻市委的决策部署，今年重点在以下几个方面要改革突破。一是制订新区产业投资负面清单，推行“非禁即入”。二是开展企业投资项目“零审批”“零收费”试点，并及早在全市推行。三是改革商事登记制度，使企业设立时间缩短为内资2天、外资3天，努力成为全国企业登记设立时间最短的地区之一。四是创新投融资体制，积极引进保险、证券、基金、融资租赁等金融机构，扩大资本市场融资规模，推进企业上市和国企改革步伐，鼓励各类社会资本投资新区开发建设。五是完善港口岸线、海域使用权统一收储制度和市场化配置机制。六是深化市场监管、陆海综合执法等行政执法体制改革。七是创新基层社会治理机制，探索建设新型社区服务中心。八是建立“生态新区、美丽海岛”建设机制。九是建立完善城乡居民养老保险标准增长机制。十是构建中高职一体化的新区职业教育体系。

加强海洋科技创新。加快建设海洋科学城，推进大甬东区块开发。坚持产学研结合，发挥省海洋开发研究院等作用，建设一批工程技术中心、重点实验室、科技企业孵化器，力促国家海洋二所科考保障基地、省海洋地质勘探大队等国家级研究勘测机构落户。完善以企业为主体的技术创新体系，培育一批科技型企业，力争技改投入达到110亿元。加强人才工作，优化人才环境，继续实施新区高端紧缺人才计划，引进急需的行业领军人才、创新创业团队和高技能人才。

（四）大力推进城镇化进程，打造海上花园城。

加强城镇建设管理。树立“全域城镇化”的理念，优化海上花园城布局和形态，开展本岛南部滨海带等重点区块城市设计，规划新城、定海、普陀等生态湾区，编制勾山、甬东等控制性详规，修编城市绿化、排水等专项规划。提高城镇化建设水平，加快新城建设和定海、沈家门老城区有机更新，推进高亭、菜园美丽县城和六横小城市建设，推进长峙岛、鲁家峙综合开发。建设科创园综合体、新城媒体创意中心、定海东门车站交通综合体、半升洞区块改造提升等项目。加强城镇精细化管理，深化“三改一拆”，全面开展无违建县（区）创建工作，确保岱山、嵊泗建成无违建县。深化交通治堵，加快地下空间开发利用，建设东山隧道复线等一批桥隧工程。提升岛际客运服务水平，发展本岛城乡一体的公共交通体系，推进公交场站建设，完善公共自行车系统。

加快城乡统筹发展。联动推进新型城市化和新渔农村建设，在本岛率先形成以城带乡、城乡一体的新型城乡关系。进一步提升白泉等6个省级中心镇建设水平，完善基础设施和公共服务配套，促进人口集中、要素集聚和资源共享，努力将中心镇建设成为城乡融合发展的重要节点。加大公共财政对渔农村的投入，深入推进村庄示范整治工程，建设12个美丽海岛特色社区，推进农房集聚建设，改造农村危旧房600户。建设集体资产产权登记与交易平台，稳妥推进渔农民宅基地置换流转，实施4个城中村综合改革试点，争取在渔农村集体产权、渔农业经营体制以及农村承包地、宅基地制度改革上取得突破，推进渔农业转移人口市民化。

建设“生态新区，美丽海岛”。以“清河净水”工程为抓手，全面推进“五水共治”，建立“河长制”管理建设机制。全市集中22亿资金，加快白泉、临城、朱家尖等主要流域河道综合整治，提高蓄水和防洪排涝能力；加强水环境治理和饮用水源保护，建成岛北、平阳浦、小高亭等深度水处理工程，开工迁建虹桥水厂；推进老城区污水管网改造，完善本岛污水一体化收集处理系统，新建小干二期、干石览区块等污水处理厂，新增污水治理受益渔农户1万户；继续实施一批水源工程和岛际引水项目，大力发展海水淡化。节约集约利用土地，盘活存量建设用地，促进土地的高效利用。推进海域海岛整治修复，大力整治非法排污、违法采砂、违规捕捞、破坏岛礁资源等行为，建设海洋公园。巩固省级森林城市创建成果，实施林地彩化工程，启动建设金塘至朱家尖生态廊道。强化节能减排刚性约束，实行排污许可证管理，严格主要污染物排放浓度和总量“双控”，防治大气污染和土壤污染。开展全国节水型社会建设等试点，深入推进国家生态文明示范区试点城市和国家环保模范城市创建。

（五）努力发展社会事业，切实保障和改善民生。

加快社会事业发展。争创教育基本现代化市，推动义务教育均衡发展，实施学前教育普惠化三年提升行动，努力解决义务教育阶段外来务工人员随迁子女入学问题。进一步完善分级办学的管理体制。支持浙江海洋学院发展，做强做精国际海运学院，加快浙江大学海洋学院、舟山技师学院、绿城国际学校建设，创办新区旅游与健康职业学院。深化群众性精神文明创建活动，巩固国家卫生城市创建成果，力争全国文明城市提名权。创建公共文化服务示范区，抓好海洋数字图书馆等建设，启动市少体校迁建、市体育场、省海洋运动基地等工程，抓好一批精品赛事活动。深化基层医疗卫生综合改革，推进市级优质医疗资源下沉，全面开展社区全科医生签约服务。实行公立医院法人治理结构改革，探索管办分离、政事分开。推进市中心血站、市精神病医院等建设。完善海岛特殊生育政策，促进优生优育和人口均衡发展。支持驻舟部队建设，抓好国防动员和后备力量建设，深化建设军民融合发展示范区。

提升社会治理水平。深化“网格化管理、组团式服务”，开展社会组织公共服务示范点建设。深化基层平安创建活动，完善矛盾纠纷大调解机制，加强流动人口和特殊人群服务管理，改革信访工作制度。创新劳动关系协调机制，推进和谐劳动关系建设。严格落实安全生产责任制，加强油品储运、无动力船舶、海洋渔业、宗教场所等安全管理，完成水产制冷行业综合整治任务。依法管理网络，正确引导网络舆论。构建立体化社会治安防控体系，依法严厉打击各类犯罪活动，营造良好的新区发展环境。

加强民生保障。以高校毕业生、城镇下岗失业人员、渔农村劳动力等为重点，加大就业援助力度，推动实现更高质量的就业，努力实现居民收入增长与经济发展同步。推进被征地农民养老保障和事业单位职工养老保险制度改革，深化研究老年渔民养老保障问题，城乡居民基础养老金待遇水平提高20%。扩大民办医疗机构纳入医保定点范围。推进养老服务体系建设，建成市社会福利中心二期等项目，扶持民办养老机构。支持慈善事业发挥济贫救困的积极作用。推进红十字公益性应急救护培训，不断提升居民自救互救能力。

今年要继续抓好十方面为民实事项目。一是实施“清河净水”工程。大陆引水二期全面供水；完成2个水厂深度水处理改造任务；城市生活污水集中处理率达到85%以上；完成90个行政村的渔农村生活污水治理；消灭垃圾河，改善黑臭河水质，完成主流域50公里河道的改造提升。二是推进“菜篮子”工程。开设水产品直供点10个；新建市级蔬菜基地2000亩；完成新区三农农副产品综合批发市场主体工程；改造提升农贸市场5家、社区菜店15家。三是深化安居工程建设。新开工各类保障性住房8900套，竣工5600套，交付3500套。四是努力促进城乡就业。全市新增城镇就业人员9000人，引导和帮助4000名城镇失业人员实现再就业；对有就业培训需求的渔农民，提供“订单式”免费培训。五是加大社会保障和社会帮扶力度。农村低保标准提高到城镇水平的90%；建成城乡社区居家养老服务照料中心20家，转型升级敬老院5家；为1000名残疾人提供助听、助行、助明服务。六是完善公共文化体育服务体系。完成舟山博物馆新馆布展并免费向社会开放；具备条件的城区内公办学校室外体育设施，非教学时间免费向社会开放；建成村级文化礼堂30个。七是积极推进教育公平。全市公办普通高中取消“三限”招生，取消择校收费；选派50名骨干教师充实到薄弱学校和农村学校，选派60名市级名师结对帮扶薄弱学校和农村学校的中青年教师。八是加大医疗惠民力度。在全市公立医疗机构开展预约转诊挂号，市级医院专家门诊预约挂号率40%以上；设置50家远程医疗会诊服务点，远程医疗服务覆盖人口达80%以上。九是强化食品药品安全监管。整合建设食品药品检测中心；全市所有社区（村）均配备食品安全协管员；改造农贸市场食品质量快速定性检测点10个，新增设14个，配置率达到60%。十是推进交通惠民、深化交通治堵。建成城市绿道15公里；本岛公交车通村率达100%，城市公交分担率提高2个百分点；本岛范围（含朱家尖）实行1小时内所有公交线路双向免费换乘；完成通村联网公路40公里；新增公共停车位4000个。

三、全面加强政府自身建设

推进新区建设，需要我们解放思想，敢为人先，干出新区地位；需要我们能快则快，又好又快，干出新区速度；需要我们攻坚突破，敢打硬仗，干出新区气势。

加快转变政府职能。坚持“该管的管到位、能放的放到底”，不断提高政府的市场意识和服务新区发展的能力。深化行政审批制度改革，推行权责清单制度，全面承接省级经济管理权限，继续精简和下放市级审批事项。完善新区机构改革，健全部门运行机制。完善财政政策，集中财力办大事，提高专项资金使用绩效，完善市与区、功能区的财政体制。出台鼓励社会力量举办教育、医疗、养老等公共事业的政策。

切实改进工作作风。认真开展党的群众路线教育实践活动，积极查摆和解决“四风”突出问题，努力建设服务型政府。倡导“只有日期没有星期，只有工作日没有休息日”的工作精神，敢于担当，不断提高政府执行能力。实行项目和工作包干制，切实解决推诿扯皮和“中梗阻”现象，确保政令畅通、令行禁止。

不断提升能力水平。适应新区加快发展新形势，建设学习型政府，打造高素质公务员队伍，学习新知识，提高推进规划实施、项目落地、工作落实的能力。研究新情况新问题，积极推进政府工作创新、制度创新、政策创新，努力在破解重点难点问题上求突破、见实效。

大力加强廉政建设。提高依法行政能力，认真执行人大及其常委会的决议和决定，支持政协发挥政治协商、民主监督、参政议政的作用，努力建设法治政府。坚持用制度管人、管权、管事，健全廉政风险防控机制，强化对招投标、土地出让、政府采购等的监管，严肃查处各类违法违纪案件，坚决防止和纠正损害群众利益的不正之风，努力建设廉洁政府。坚决压缩“三公”经费支出，确保在去年预算基础上缩减30%。

各位代表：雄关漫道真如铁，而今迈步从头越。让我们更加紧密地团结在以习近平同志为总书记的党中央周围，在省委、省政府和市委的坚强领导下，紧紧依靠全市人民，解放思想，凝心聚力，勇担使命，四干兴舟，不断开创浙江舟山群岛新区建设新局面！
